# Pathophysiology of Mitochondrial Dysfunction in Human Spermatozoa: Focus on Energetic Metabolism, Oxidative Stress and Apoptosis

**DOI:** 10.3390/antiox10050695

**Published:** 2021-04-28

**Authors:** Chiara Castellini, Settimio D’Andrea, Giuliana Cordeschi, Maria Totaro, Antonio Parisi, Giovanna Di Emidio, Carla Tatone, Sandro Francavilla, Arcangelo Barbonetti

**Affiliations:** Andrology Unit, Department of Life, Health and Environmental Sciences, University of L’Aquila, 67100 L’Aquila, Italy; chiara.castellini@univaq.it (C.C.); settimio.dandrea@graduate.univaq.it (S.D.); giuliana.cordeschi@univaq.it (G.C.); maria.totaro@graduate.univaq.it (M.T.); antonio.parisi1@graduate.univaq.it (A.P.); giovanna.diemidio@univaq.it (G.D.E.); carla.tatone@univaq.it (C.T.); sandro.francavilla@univaq.it (S.F.)

**Keywords:** adenosine trisphosphate (ATP), apoptosis, glycolysis, mitochondria, oxidative phosphorylation, spermatozoa, energetic metabolism, sperm motility, reactive oxygen species (ROS)

## Abstract

The dogma of mitochondria as the major source of energy in supporting sperm motility should be critically reconsidered in the light of several experimental data pointing to a major role of glycolysis in mammalian spermatozoa. In this light, the reported positive correlation between the mitochondrial membrane potential (ΔΨm) and motility of ejaculated spermatozoa cannot be explained convincingly by an impaired mitochondrial ATP generation only. Evidence has been produced suggesting that, in human sperm, dysfunctional mitochondria represent the main site of generation of reactive oxygen species (ROS). Furthermore, in these organelles, a complex bidirectional relationship could exist between ROS generation and apoptosis-like events that synergize with oxidative stress in impairing sperm biological integrity and functions. Despite the activity of enzymatic and non-enzymatic antioxidant factors, human spermatozoa are particularly vulnerable to oxidative stress, which plays a major role in male factor infertility. The purpose of this article is to provide an overview of metabolic, oxidative and apoptosis-like inter-linkages of mitochondrial dysfunction and their reflections on human sperm biology.

## 1. Introduction

Mitochondria are sub-cellular organelles of elliptical shape, consisting of an outer membrane and an inner membrane separated by an intermembrane space. The inner mitochondrial membrane folds to form cristae extending into a protein-dense matrix which contains mitochondrial DNA (mtDNA). The existence of mtDNA, together with structural homologies of the inner membrane with the prokaryotic cell membrane, has led to hypothesize that mitochondria were once bacteria that invaded eukaryotic cells, establishing a symbiotic relationship. This symbiosis would be evolved into a more complex organism, acquiring the ability to generate energy more efficiently than glycolysis using aerobic metabolism [1].

Oxidative phosphorylation (OXPHOS) requires the coordinated activity of the electron transport chain (ETC) and adenosine trisphosphate (ATP) synthase, both located in the inner mitochondrial membrane, and produces approximately 90% of cellular energy [2]. Nevertheless, in many species, including mice [3,4] and humans [5,6,7], glycolysis would be used as a preferential pathway to synthesize ATP for maintaining sperm motility.

Besides the ATP-generating activity, mitochondria also play key roles in controlling the sperm lifespan, since they represent an interplay center between the generation of reactive oxygen species (ROS) [8,9] and the activation of molecular pathways leading to apoptosis-like changes [10].

This review summarizes current knowledge on the molecular reflections of mitochondrial dysfunction on sperm biology, focusing on the role of mitochondria in sperm energetic metabolism and oxidative/apoptotic events.

## 2. Are Mitochondria Really the Energetic Motor of Mammalian Sperm?

The role of mitochondria in the energetic support of sperm motility is a matter of debate [11,12,13]. Two pathways can account for the generation of ATP in mammalian spermatozoa, glycolysis and mitochondrial respiration. As mitochondrial OXPHOS is much more efficient than glycolysis in generating ATP, it has been widely accepted that the ATP needed for sperm motility is synthesized by mitochondrial respiration.

In mammalian spermatozoa, mitochondria rearrange in tubular structures that are helically distributed around the anterior portion of the axoneme, constituting the midpiece [14,15]. As the sperm flagellum is long and thin and mitochondria are confined in its proximal end, the question has been raised as to whether OXPHOS-derived ATP can passively diffuse through the entire flagellum to efficiently support axoneme activity. In sea urchin sperm, a shuttle mechanism to facilitate the ATP diffusion along the flagellum is provided by the creatine phosphate (CrP) that buffers the ATP/adenosine diphosphate (ADP) ratio at the expense of CrP/creatine [16]. However, mammalian spermatozoa lack or contain only low amounts of CrP or other phosphagens [17,18], making it unlikely that the CrP shuttle plays a major role in providing ATP from mitochondria to the axoneme. Indeed, spermatozoa from knockout mouse models where the gene for the mitochondrial isotype of creatine kinase had been inactivated exhibited similar motility patterns to the wild-type controls [19]. These legitimate considerations shifted the focus from OXPHOS to glycolysis.

Although mitochondrial respiration is more efficient than glycolysis in generating ATP molecules, key enzymes of glycolysis are tethered to the fibrous sheath of the principal piece [20,21,22,23], and hence they might assure an efficient production of ATP for dynein ATPase locally in the entire length of the flagellum. Consistent with this view, in mouse [3], bovine [24] and human spermatozoa [5,6,7], motility was not affected by mitochondrial inhibition when glucose was available in the extracellular medium. We previously demonstrated that in a medium lacking glycolysable sugars, the presence of substrates for OXPHOS such as pyruvate and lactate fully supported the motility of human spermatozoa [7]. Interestingly, under such experimental conditions, the addition of 2-Deoxy-D-glucose (DOG), which inhibits glycolysis by competing with glucose for key enzymes, significantly decreased sperm motility [7]. This evidence was incompatible with the hypothesis that ATP is synthesized in mitochondria and then provided to the entire axoneme by diffusion. On the contrary, these findings supported the notion that ATP produced by OXPHOS is used to drive gluconeogenesis and thus to supply glucose to glycolytic enzymes for ATP production in the principal piece.

In this light, glycolysis would compensate for any lack of ATP production by mitochondria in maintaining sperm motility, and mitochondrial OXPHOS inhibition could depress motility only under experimental conditions of concomitant glycolysis blockage. However, differences among the species exist, as stallion spermatozoa rely primarily on mitochondrial respiration to generate energy required for motility [25]. Overall, it is conceivable that both glycolysis and OXPHOS contribute to ATP production, depending on each other in controlling sperm functions according to the different availability of energetic substrates in the environment [4]. Of note, in female genital tract fluids, the concentrations of lactate are higher than those of glycolysable substrates [26,27,28,29], suggesting a possible major role of mitochondrial respiration in supporting sperm motility. This hypothesis could explain why spermatozoa retain a high number of mitochondria during their differentiation, despite the dramatical decrease in the cellular volume resulting from the removal of any unnecessary structure. Anyway, an obligatory role for glycolysis seems to be confirmed by the loss of progressive motility in spermatozoa of mouse models where the gene for sperm-specific glyceraldehyde-3-phosphate dehydrogenases had been knocked out [4]. In this view, the reported correlation of the mitochondrial membrane potential (ΔΨm) [30] or mitochondrial morphologic integrity [31] with the motility of ejaculated spermatozoa cannot be explained convincingly by an impaired mitochondrial ATP generation only.

Noteworthy, in human spermatozoa, a mitochondrial dysfunction could affect motility when it is accompanied by an intrinsic generation of ROS. Oxidative stress, indeed, is responsible for membrane lipid peroxidation [5,32] and promotes the activation of mitochondrial pathways resulting in apoptosis-like changes.

## 3. Biochemistry of Reactive Oxygen Species: An Overview

**Figure 1:**
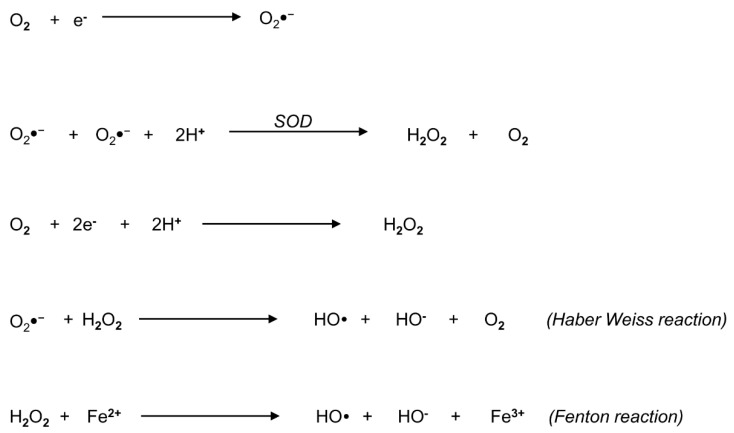
Biochemical overview of reactive oxygen species (ROS) and their generating reactions. The metabolism of oxygen generates a widespread group of molecules, comprehensively called ROS, that include free radicals and peroxides. The principal form of ROS is the superoxide anion radical (O_2_^●−^), which can be generated by univalent reduction of oxygen (O_2_) through the mitochondrial electron transport chain. The radical O_2_^●^^−^ is converted into hydrogen peroxide (H_2_O_2_) according to two different pathways: in the first pathway, superoxide dismutase (SOD) catalyzes the production of H_2_O_2_ and O_2_; the second pathway involves the bivalent reduction of O_2_. The Haber–Weiss reaction between H_2_O_2_ and O_2_^●−^ generates the hydroxyl radical (OH) and O_2_. Finally, in the Fenton reaction, H_2_O_2_ decomposes into OH in the presence of ferrous ion.

Reactive oxygen species represent a widespread group of molecules that include free radicals and peroxides, produced from the metabolism of oxygen (Figure 1).

Examples of non-radical ROS include hydrogen peroxide (H_2_O_2_), while superoxide anion (O_2_^●−^), the hydroxyl radical (OH) and the hydroperoxyl radical (HO_2_) are free radicals, where an oxygen molecule contains one or more unpaired electrons in its molecular orbital [33]. Other powerful oxidants also include molecules derived from the reaction of oxygen with carbon-centered radicals, such as peroxyl radicals (ROO), organic hydroperoxides (ROOH) and alkoxyl radicals (RO) [33]. In particular, O_2_^●−^ represents the principal form of ROS that can be accidentally generated by univalent reduction of oxygen through the mitochondrial ETC [34]. It can react with biological tissues, but its toxicity is low and widely related to its conversion into H_2_O_2_, according to different pathways (Figure 1): the first one is the reaction catalyzed by superoxide dismutase (SOD), producing H_2_O_2_ and oxygen; the second pathway is the bivalent reduction of oxygen [33]. A reaction between H_2_O_2_ and O_2_^●−^ generates OH, which may also originate from H_2_O_2_ in the presence of a ferrous ion promoter by the Fenton reaction [33].

## 4. Origin of ROS in Semen

Within semen, ROS can be generated by two main sources: leukocytes (extrinsic ROS) and spermatozoa themselves (intrinsic ROS) [35].

Almost every semen sample contains leukocytes, particularly macrophages and neutrophils [36]. Seminal leukocytes have the potential to promote oxidative stress since they destroy pathogens mainly generating ROS. Although seminal white cells produce ROS with a rate 1000 times higher than spermatozoa [37], subclinical concentrations (<1 × 10^6^/mL) of seminal leukocytes seem to not be harmful to sperm quality [38]. This may be because seminal neutrophils originate from secondary sexual glands; therefore, they make contact with sperm only at the ejaculation time, when antioxidant factors of seminal plasma can preserve spermatozoa from oxidative damage. Indeed, even the association of leukocytospermia (≥1 × 10^6^ white cells/mL of semen) with male fertility is still under debate. In a recent meta-analysis of case–control studies, we recently demonstrated that in men seeking consultation for couple subfertility, the presence of leukocytospermia is not associated with poorer outcomes of assisted reproductive technology (ART) or poorer semen quality in populations asymptomatic for genital tract infections [39].

**Figure 2:**
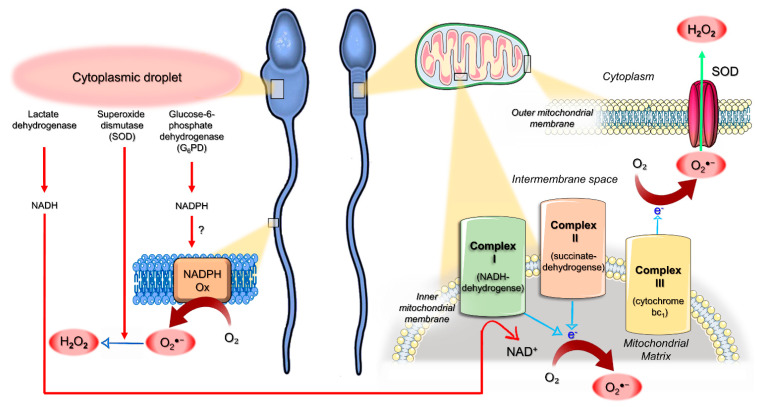
Schematic model for the origin of reactive oxygen species (ROS) in human spermatozoa. Cytoplasmic droplets of immature teratozoospermic spermatozoa are rich in glucose-6-phosphate dehydrogenase (G_6_PD), an enzyme involved in the intracellular production of the reduced form of nicotinamide adenine dinucleotide (NAD) phosphate (NADPH), which could represent the substrate for the generation of the superoxide anion radical (O_2_^●−^) by NADPH oxidase systems (NADPH Ox). Superoxide dismutase (SOD) and lactic acid dehydrogenase are also present in cytoplasmic droplets. While the former generates H_2_O_2_ from O_2_^●−^, the latter produces the reduced form of NAD (NADH), which undergoes oxidation by mitochondrial complex I. Within mitochondria, a small amount of the oxygen (O_2_) reduced during mitochondrial oxidative phosphorylation (OXPHOS) undergoes univalent reduction, thus generating O_2_^●−^. This radical can be metabolized by spontaneous or SOD-mediated dismutation in H_2_O_2_ or can readily move into the cytoplasm via voltage-dependent anion channels. However, an increased NADH synthesis by the lactic acid dehydrogenase (e.g., in the presence of a cytoplasmic droplet) can overcharge complex I, raising the rate of O_2_^●−^ generation. Dysfunctional mitochondria of defective sperm from infertile men can display high rates of univalent O_2_ reduction, resulting in high rates of O_2_^●−^ generation, irrespective of the presence of cytoplasmic droplets (see the text for details). In complex I and complex III, electrons can directly react with O_2_: while O_2_^●−^ generated by complex III is released in the intermembrane space and rapidly dismutated to H_2_O_2_ in the cytoplasm, complex I generates O_2_^●−^ that is directly released into the mitochondrial matrix, thus hindering the escape and promoting oxidative stress.

Spermatozoa themselves represent another source of ROS (Figure 2). They exhibit both the mitochondrial nicotinamide adenine dinucleotide (NAD)-dependent oxidoreductase [40] and the membrane calcium-dependent NAD phosphate (NADP) oxidase (NOX5) system [41]. The latter generates small and controlled amounts of O_2_^●−^, playing a role in controlling human sperm motility under physiological conditions [41]. Immature teratozoospermic spermatozoa often display cytoplasmic droplets that are rich in glucose-6-phosphate dehydrogenase (G_6_PD), an enzyme involved in the intracellular production of the reduced form of NADP (NADPH). Therefore, it has been hypothesized that retention of the residual cytoplasm by spermatozoa is positively correlated with ROS generation via mechanisms that could be mediated by G6PD activity (Figure 2) [42]. Cytoplasmic droplets also contain SOD and lactic acid dehydrogenase [35]: SOD generates H_2_O_2_ from O_2_^●−^; meanwhile, lactic acid dehydrogenase produces the reduced form of NAD (NADH). The oxidation of NADH at the mitochondrial complex I activates the ETC, ultimately leading to the bivalent reduction of oxygen and ATP generation [43,44]. The ETC is composed of respiratory enzyme complexes organized in the inner mitochondrial membrane and includes: NADH-dehydrogenase (complex I), succinate dehydrogenase (complex II), cytochrome bc1 (complex III) and cytochrome oxidase (complex IV). Within mitochondria, 1–2% of the oxygen reduced during OXPHOS undergoes a univalent reduction, thus generating O_2_^●−^ [45]. O_2_^●−^ can be metabolized by spontaneous dismutation or by mitochondrial SOD in H_2_O_2_ or can readily move into the cytoplasm via voltage-dependent anion channels [46]. However, an upstream increase in the synthesis of NADH by the lactic acid dehydrogenase (e.g., in the presence of a cytoplasmic droplet) can overload ETC complex I, thus increasing the rate of O_2_^●−^ generation. Actually, dysfunctional mitochondria of defective spermatozoa from infertile men can display high rates of univalent oxygen reduction, resulting in O_2_^●−^ generation, irrespective of the pathways related to the retention of the residual cytoplasm. The principal sites of mitochondrial ROS production are complex I and complex III, where electrons can directly react with oxygen or other electron acceptors [5]. The radical O_2_^●−^ which is generated by complex III is released in the intermembrane space where it is rapidly dismutated to H_2_O_2_ in the cytoplasm (Figure 2), thus escaping to the extracellular space [5]. On the contrary, O_2_^●−^ generated by complex I is directly released into the mitochondrial matrix (Figure 2), where the escape is hindered: when the production of O_2_^●−^ in the mitochondrial matrix had overwhelmed the intramitochondrial antioxidant defenses, oxidative stress occurred [5]. The mitochondrial generation of O_2_^●−^ can be revealed using MitoSOX-Red, a lipid-soluble cation that selectively targets mitochondria: it is rapidly oxidized by O_2_^●−^ only and fluoresces red upon binding to nucleic acid [5].

Mitochondria-generated ROS trigger lipid peroxidation, which can be assessed at flow cytometry by BODIPY C_11_. This probe is readily incorporated into biologic sperm membranes and responds to free radical attack with a spectral emission shift from red to green [47]. Lipid peroxidation reactions culminate in the generation of aldehydes, which can covalently bind cysteine, lysine and histidine residues on target mitochondrial proteins [48]. These adducts dysregulate the electron flow through the ETC, further increasing the rate of O_2_^●−^ generation in a self-perpetuating cycle [48]. An interesting mechanism underlying pro-oxidative mitochondrial dysfunction involves the inhibition of key enzymatic antioxidants termed peroxiredoxins (PRDXs). PRDXs display efficient scavenging activities against a wide variety of ROS [49,50,51] and represent a major first-line defense of human spermatozoa against oxidative stress [50,52]. Although PRDXs are largely distributed in seminal plasma as well as in all subcellular sperm compartments [53], spermatozoa from infertile men exhibit lower amounts of PRDX1 and PRDX6 with a relatively high degree of thiol oxidation [54] which inhibits PRDX activities [50,51,53,55]. In particular, the inhibition of the calcium-independent phospholipase A_2_ (Ca^2+^-iPLA_2_) activity of PRDX6 promotes a dysregulation of mitochondrial function accompanied by an increased mitochondrial generation of O_2_^●−^ [52,56]. The resultant high levels of 4-Hydroxynonenal (4HNE), an end product of lipid peroxidation, will further contribute to mitochondrial dysfunction, ultimately leading to DNA oxidation, as revealed by the generation of the oxidized base adduct 8-hydroxy-2′-deoxyguanosine (8-OHdG) [48].

Of note, a complex bidirectional relationship could exist between ROS generation and the activation of intrinsic (mitochondrial) apoptosis-like pathways that synergize with oxidative stress in impairing sperm biological integrity and functions.

## 5. Mitochondria as an Interplay Center between Oxidative Stress and Apoptotic Events

In somatic cells, the mitochondrial (intrinsic) pathway of apoptosis is triggered by proapoptotic BH3 proteins, which can be activated by noxious stimuli, including ROS, γ radiations and DNA injuries [57]. These proteins inhibit antiapoptotic BCL2-BCL-XL, thus alleviating the inhibition of proapoptotic factors BAX and BAK. The BAX/BAK-mediated increase in mitochondrial permeabilization results in the loss of ΔΨm and cytochrome c release from the inter-mitochondrial membrane space into the cytosol [58,59,60,61]. The cytochrome c release leads to the activation of caspase 9 through the apoptosome [57]. Activated caspase 9 ultimately activates caspase 3, the downstream “executioner” caspase, a protease that promotes both the cleavage of cytoskeletal proteins and the activation of DNases [60,62].

Interestingly, apoptosis-like features can be induced also in human spermatozoa by exposure to a variety of non-receptor-mediated stimuli, ultimately leading to oxidative stress, including cryostorage [63], exposure to radiofrequency electromagnetic radiation [64] and direct addition of H_2_O_2_ [65]. It has been demonstrated that a key event promoting this truncated (mitochondrial) apoptotic cascade would be the inhibition of the phosphoinositide 3-kinase (PI3K) signaling pathway [10]. This finding extends the spectrum of the PDXs tasks to the apoptosis control, as PDX6 Ca^2+^-iPLA_2_ activity plays a pivotal role in maintaining the phosphorylated (active) status of PI3K, thus preserving sperm survival [66].

Noteworthy, while ROS can be considered a trigger for the mitochondrial pathway of apoptosis, at the same time, the latter is associated with oxidative stress, consequently establishing a vicious cycle. As demonstrated in human somatic cell lines, the release of cytochrome c into the cytosol represents a pro-oxidative event because it is associated with ETC disruption, leading to increased O_2_^●−^ generation [67]. This might explain the concomitant occurrence of mitochondrial ROS generation and caspase-9-related apoptosis-like changes in human sperm under certain experimental conditions promoting the loss of ΔΨm [32,68].

## 6. Pathophysiology of Oxidative Stress in Human Spermatozoa

Low levels of ROS are normally produced by human spermatozoa and are involved in sperm physiological processes, such as tyrosine phosphorylation and sperm hyperactivation during capacitation, acrosome reaction and sperm–oocyte interactions [58,60,69]. Nevertheless, an abnormal and uncontrolled increase in ROS generation exerts detrimental effects on sperm biology, resulting in membrane and genomic damages [59,60].

Among free radicals, ^●^OH is the most reactive molecule due to its unpaired electron, leading to the oxidation of lipids in biological membranes, amino acids in proteins and carbohydrates within nucleic acids. Membrane lipid peroxidation is initiated by the abstraction of a hydrogen atom from membrane fatty acids, resulting in the generation of a carbon-centered radical which rapidly reacts with oxygen. The resulting lipid peroxyl radical, in order to stabilize, abstracts a hydrogen atom from an adjacent fatty acid, generating another carbon-centered radical, which propagates the chain reaction within the membrane. This process produces cytotoxic adducts, such as malondialdehyde, acrolein and 4HNE, affecting membrane fluidity and fusogenicity, which are required for motility, acrosomal exocytosis and sperm–oocyte interaction [60,61,62]. Moreover, a self-perpetuating cycle of ROS production is triggered when lipid aldehydes bind to mitochondrial ETC proteins [33]. Although intracytoplasmic sperm injection (ICSI) can overcome the consequences of membrane damages, the oxidation of purine and pyrimidine bases and the deoxyribose backbone impairs sperm DNA integrity, compromising both the viability of and paternal genomic contribution to the embryo [59,61]. In particular, ROS delivered from sperm mitochondria can rapidly move from the midpiece to the sperm head, thus oxidizing the DNA.

Indeed, human spermatozoa are particularly vulnerable to oxidative stress due to the cellular organization and biochemical factors. The high content of polyunsaturated fatty acids (PUFA) in the sperm membrane increases the susceptibility to membrane lipid peroxidation. Polyunsaturated fatty acids are involved in maintaining the physiological fluidity and fusogenicity of sperm membranes, but, unfortunately, PUFA are also particularly vulnerable to free radical attack because of the lowest carbon–hydrogen dissociation energies at the bisallylic methylene position. During spermatogenesis, spermatozoa lose most of their cytoplasm that in somatic cells contains efficient antioxidant enzymes [66,70,71,72]. Although seminal plasma and all subcellular sperm compartments contain PRDXs, ensuring an effective first-line defense against ROS [50,52], spermatozoa from infertile men display lower amounts of PRDX1 and PRDX6 with a relatively high degree of thiol oxidation [54]. Seminal plasma also contains several non-enzymatic antioxidant factors, including ascorbic acid, glutathione, albumin, α-tocopherol, carnitine, amino acids, flavonoids and carotenoids [35], that can execute their activity by two main mechanisms. First, they can chemically neutralize free radical activity in a direct way; secondly, they can become oxidized themselves, like albumin [68]. Moreover, metal chelators of seminal plasma, such as lactoferrin, transferrin and ceruloplasmin, are also able to block the ROS generation [69]. However, during the epididymal transit and within the female genital tract, spermatozoa have no contact with seminal plasma and its antioxidant factors: this could make sperm more vulnerable to oxidative stress damages, especially in the presence of genital tract infections.

## 7. Conclusions

The notion that mitochondria represent the motor of mammalian spermatozoa is changing in the light of increasing evidence pointing to glycolysis as the preferred metabolic pathway for the energetic support of sperm motility in many species. Available data indicate that both glycolysis and mitochondrial respiration would contribute to ATP production, depending on each other in controlling sperm functions according to the availability of environmental energetic substrates. However, irrespective of the metabolic reflections, dysfunctional mitochondria would play a pivotal role in influencing sperm survival, representing the key cellular organelles for the interplay between ROS generation and intrinsic apoptosis-like events. A better understanding of these processes could represent the basis for developing mitochondria-centered antioxidant molecules to improve sperm functions and male reproductive potential.

## Data Availability

No new data were created or analyzed in this study. Data sharing is not applicable to this article.
